# Relationship between the weights of seed beetles of the genus *Megacerus* Fåhraeus, 1839 (Coleoptera: Chrysomelidae: Bruchinae) and their host seeds of the family Convolvulaceae

**DOI:** 10.1038/s41598-019-44761-8

**Published:** 2019-06-11

**Authors:** A. Canto, R. Rodríguez, E. Reyes-Novelo

**Affiliations:** 1 Centro de Investigacion Cientifica de Yucatan, A.C., Merida, Mexico; 2 Centro de Investigaciones Regionales Dr. Hideyo Noguchi, Universidad Autonoma de Yucatan, Merida, Mexico

**Keywords:** Biodiversity, Community ecology

## Abstract

We studied seeds from a set of plant species from the Convolvulaceae family. Seeds collected from natural populations and infested with beetles of genus *Megacerus* were monitored until the beetle emergence. We analyze the relationship between body weight of beetles and seed weight of host plants, and its connection with between-species differences and sexual dimorphism. The results show that differences in the scaling of body weight of beetles are associated with sexual dimorphism. For the same species of beetle, the females tend to have heavier bodies than the males. Differences between host plants species in the weight of seeds are related to differences in the body weight *Megacerus* species, resulting in a distinctive pattern of seed infestation across hosts. Small-sized (lighter) species of beetles tended to infest small-sized (lighter) seed species and, correspondingly, heavier beetles species tended to do it in heavier seed species. Mechanisms of female oviposition preferences may be involved to generate that pattern. In general, the beetle weight showed an asymptotic relation with the host seed weight. The greater the weight of the seed, the greater the weight of adult beetle was. However, the proportion in weights reaches an asymptotic value probably because beetles reached the maximum possible weight for their species. We conclude that the process of specialization in the seed-beetle assemblage studied is influenced by intrinsic traits of the species involved in the interaction (beetles and seeds) and by differences between sexes in their sexual-allocation paths.

## Introduction

In the seed-beetle interactions, the size of the seed is a direct determinant of the quality of the adult beetles. The availability of food within the seeds is essential for the adaptation of individuals since the acquisition of nutrients during the larval stages defines the beetles’ performance in adult life^1,2^.

Seeds have an embryo, cotyledons and endosperm that are rich in carbohydrates and proteins and together provide sufficient resources for the full development of the larvae. However, depending on the environment and natural history, seeds vary in the amount of resources they contain, both among individual plants and among species^3^, thus resulting in variations in their quality as a food source for the seed beetles^4^.

The quality of the seed components strongly influences the adaptation and reproductive strategies of the insects because the body size of the individuals is modified based on the accumulation of resources, and body size is a key feature for individual performance reflected in development and survival^5^. For example, beetles that emerge from larger seeds tend to be larger adults that survive longer, have a greater ability to evade predators and a better immune capacity than smaller-sized beetles^1,6,7^.

Variations in seed weight directly affect fertility of individuals and can lead to differentiation in size between males and females. The evolutionary specialization of the reproductive roles between males and females produces sexual size dimorphism differences regardless of the proportional effect of the seed size on body size of beetles. This is because, weight gain in females is mostly invested in fertility (to lay more eggs), while resource acquisition in males is invested mostly in structures associated with display for mating. Larger females will have higher fecundity but males with larger display structures will have more opportunity to attract females. The final result of the differential investment of resources between females and males is that females will be heavier than males regardless of the weight of the host seed^5,8,9^. Differences in the weight of seeds between the plant species that acts as the seed host is a trait that can influence the final size of the individuals^4,9^. In the present work, a particular set of species of seed beetles of the genus *Megacerus* (Coleoptera: Chrysomelidae: Bruchinae) and seeds of the family Convolvulaceae of southeastern Mexican region are studied. The larval stages of this group of beetles depend mainly on the seeds of this family of plants to complete their development^10,11^. The beetle and plant species are native to Mexico and distributed in practically all environments of the Yucatan Peninsula. Thus far, 11 *Megacerus* species have been reported in 10 plant species for the region^12,13^. The distribution of beetles follows the distribution of the host plants, which are relatively abundant species that flower simultaneously in masse during the cold season^14^.

This system of interactions between beetle and seed species is conducive to exploring the effect of the seed characteristics on the beetles that prey on different seed species since the same species of beetle can invade different seed species with different sizes and one seed species can vary in size and be invaded by different species of beetles that vary in size. Additionally, seed and beetle species can be identified relatively easily at the species level. *Megacerus* species present an evident sexual dimorphism in antennae and eyes that makes them recognizable from emergence.

The purpose of the study is to gain knowledge about the underlying ecological specialization between beetle species and their host seed species. We hypothesize that the relationship of weights between seeds and adult beetles in the Convolvulaceae-*Megacerus* interactions, is modified by ecological components such as variation in the seed weight within species of host plants and sexual dimorphism in the body size of beetles, as well as differences in the seed weight of host plant species. We expect sex-biased differences in the body weight scaling between males and females, differences in the body weight scaling within *Megacerus* species that use host seeds of different weight, and that the body weight in beetles of the same species changes proportionally to the weight of their host seed.

## Materials and Methods

### Study area

Within the Yucatan region, four areas were selected from north to south that host wild populations of the most frequent species of Convolvulaceae. The areas were Conkal (21°04′24″N; 89°31′15″W), Yaxché (21°07′02″N; 89°38′08″W), Xcunyá (21°07′58″N; 89°36′47″W) and Kiuic (20°03′05″N; 89°33′55″W), which have similar temperature and precipitation conditions, with an average monthly temperature of 26 °C and average annual precipitation of 1096 mm^15^. The predominant type of vegetation in the areas to the north is lowland deciduous forest and to the south is mid-elevation evergreen forest, and the altitude range varies from 9 masl in the northern areas to 90 masl in the southern areas^16^.

### Seed beetles

The beetles of the genus *Megacerus* are exclusive to America and belong to the monobasic tribe Megacerini within the subfamily Bruchinae associated to Convolvulaceae family^10,11,17^. They present four instars, from larvae to pupa whose development depends entirely on the seeds^18^. In the study area, larvae of the *Megacerus* species develop inside seeds of plant species of the genera *Ipomoea* and *Merremia* (Convolvulaceae). Male and female adults of *Megacerus* can often be found feeding on the nectar and pollen of the flowers of these plant genera. As far as is known, the females deposit their eggs on the immature fruits, and after few days (four to five days in *Megacerus discoidus*^18^), the larvae emerge and pierce the plant tissue until they reach the seeds and embed themselves. They then feed on the endosperm and seed embryo until reaching the imago stage (adults recently emerged from pupal cocoon).

### Plants studied

**Figure 1:**
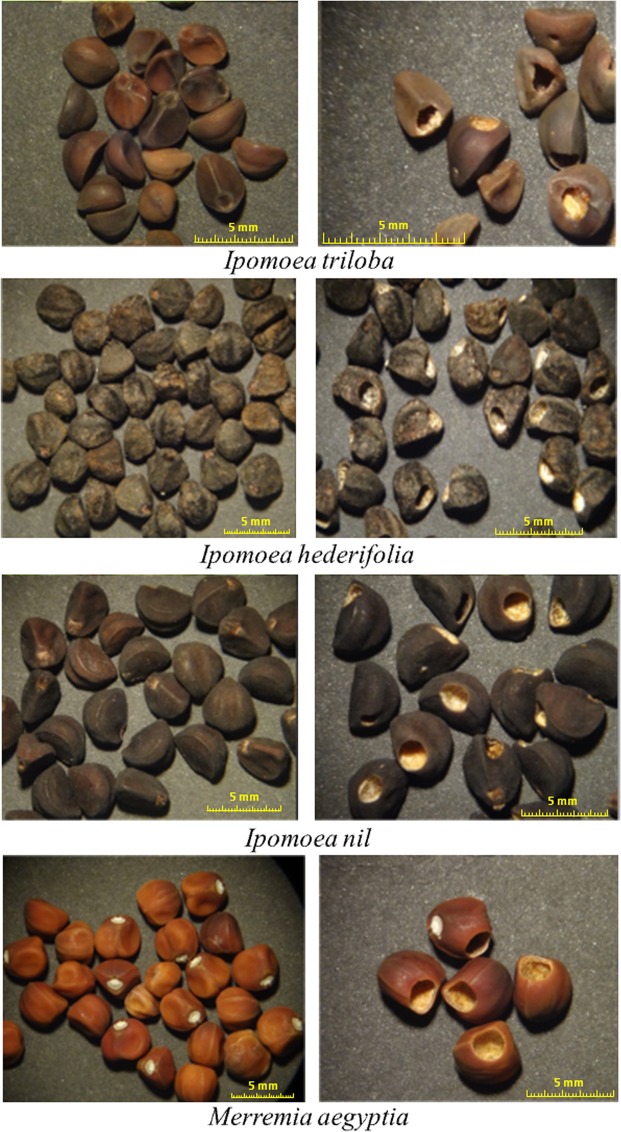
Seeds of the plant species of the Convolvulaceae family studied in the present work. Seeds without damage (left) and predated seeds (right) by beetles of the genus *Megacerus* are shown.

In each study location, 5–10 collection points were selected whose selection criterion requirements were populations of each of the following species of Convolvulaceae: *Ipomoea triloba*, *Ipomoea hederifolia*, *Ipomoea nil* and *Merremia aegyptia*. The species belong to the tribe Ipomoeeae and sister tribe Merremieae^19^, and their populations are generally found in sunny environments with open vegetation composed of herbaceous or low scrub. These plants are annual vines that grow massively and whose flowering occurs towards the cold season between September and October. The flowers are showy and hermaphroditic and have a superior ovary with two or four deeply grooved carpels, locules equal in number to carpels and four ovules in total, with two per carpel. From our field observations, the oviposition of females during the flowering season of *Merremia* and *Ipomoea* species, occurs on the external surface of the sepals of the calyx which contains the flower ovaries (the future fruit with seeds). The fruits are longitudinally dehiscent capsules in four segments with seeds that contain a cartilaginous homogeneous endosperm and a large axillary embryo that occupies most of the seed, with thin cotyledons that are extensive and folded multiple times^20^. Seed dispersal is from November to March, which is favorable for collecting the fruits since the larvae that have managed to embed themselves within the seeds are completing their development^13^. Figure 1 shows the type of seed of each species.

### Seed collection and emergence of beetles

Seed collection was carried out during three consecutive years (2008, 2009, 2010) during the seed dispersal stage, and at each collection point, 3–5 plants spatially separated by at least 20 meters were selected. From each plant, 10–12 mature completely dry fruits with fully developed seeds were collected just before dehiscence. The seeds were carefully extracted from the fruits using clean tweezers and placed individually in clean glassine envelopes, which in turn were placed in cellophane bag envelopes. The envelopes were incubated under the environmental conditions that simulated the stage of seed dispersal in their natural populations at a temperature of 25–28 °C, relative humidity of 50–60% and photoperiod of 10–12 hours of light and 12–14 hours of darkness. The newly emerged adults were immersed in 99% alcohol to facilitate their dehydration, preserved until processing, and then dried at room temperature for approximately four hours, after which they were individually weighed. The respective host seed of each beetle was subjected to an air-drying process and then individually weighed. Beetles and seeds were weighed (dry weight) with an analytical balance with an accuracy of 0.01 mg. The beetles were identified to the subspecies level and sex. A synoptic collection was selected based on external characters and then, ten individuals of each morphotype were dried and mounted in entomological pins. Male genitalia of specimens were extracted and immerse in 10% KOH solution to macerate tissues for the correct identification. The beetles were identified using the morphological criteria of Terán and Kingsolver^17^ and the taxonomical keys of Kingsolver^10,11^ and results were corroborated using the key of Romero-Napoles^21^ for Mexican *Megacerus*. All specimens were labeled and deposited in the insect collection of the Centro de Investigacion Cientifica de Yucatan (CICY).

### Data analysis

Descriptive statistic were calculated to summarize features of the collection data of body weight of individual beetles, seed weight of the host plants and the frequency of hatching of *Megacerus* species. All the analyses were carried out using the R Core Team^22^ platform and the *agricolae*^23^ module version 1.2–4 and *ggplot2*^24^ version 3.0.0. The beetle weight data were transformed to improve normality and perform the analyses adequately using the square root of the data^25^. Beetle species do not invade all seed species equally, with certain beetle species invading some seed species but not others, therefore for some combinations of seed-beetles species, there are relatively not enough data to perform robust analyses. Under this, analyses were conducted only for sets with enough data to produce robust results.

To analyze differences between females and males in the relationship between the body weight and the seed weight, a linear mixed model was fitted to the sets of *Megacerus* species with at least 10 data for each sex. The model contained the seed weight (mg) as continuous causal variable, the sex of the beetle as fixed factor and the seed weight × the sex as interaction term^25^. To test differences in the body weight of the beetle’s species that use different host seed species, a linear mixed model was used the identity of the host plant species as the main factor, and when possible, the sex of the beetle and the interaction term, the host seed species × the sex, were included as co-factors^25^. The analysis was conducted for species of beetles with at least 40 data. To calculate the F statistics in all the analyses, the sum of squares type II/III method for unbalanced data was used^26^, and the *post hoc* comparison of means between the host plant species and between sexes was carried out via Tukey’s test and t-test, respectively.

The relationship between the host seed weight and the beetle weight regardless the seed species used and the beetle species that prey on it was analyzed to have an overview of how the range of variation in the body weight match with the range of variation of the seed weight. The information will provide insights into the specialization of *Megacerus* to their host species. Three types of regression models were performed combining all the data without discriminating between the species of plants and beetles: linear, quadratic and asymptotic. The beetle weight data were transformed to their square roots to perform the analyses^25^. To determine the model that best describes the relationship between the weights, the values of the residual standard error, the goodness of fit using the coefficient of determination and the degrees of freedom between the three models were compared^27^. Biological factors of the beetle-seed system, particularly the intrinsic limits of beetle species body weight, were also considered as selection criteria for the best model.

## Results

**Figure 2:**
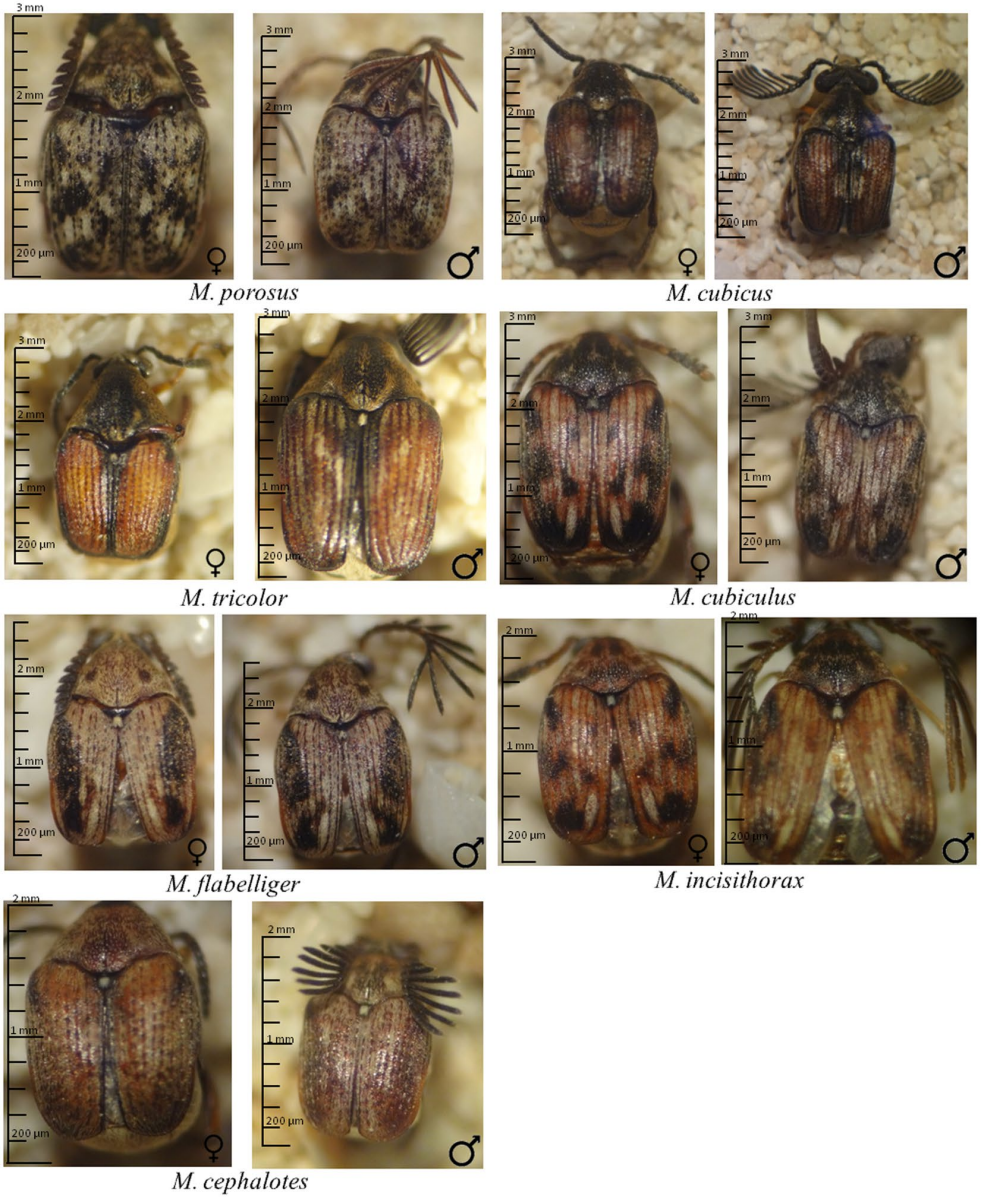
Species of beetles of the genus *Megacerus* that emerged from the seeds of plants in the family Convolvulaceae collected in this study.

Out of a total of 10510 seeds, 813 beetles emerged. All the resulting beetles belonged to the genus *Megacerus*, and seven species in total were recorded (Fig. 2). A total of 428 females and 385 males emerged from the seeds. The most frequent species of beetles was *Megacerus porosus*, followed by *M*. *cubicus*, and the less frequent species were *M*. *tricolor* and *M*. *flabelliger*. Each species of host seed received several species of *Megacerus*, with *I. triloba* receiving the fewest (3 species) and *I. hederifolia* the most (5 species). However, not all the species of beetles used several species of the host plants. *Megacerus cephalotes*, *M. flabelliger*, and *M. porosus* used the single host species, *I. triloba*, *I. hederifolia*, and *M. aegyptia*, respectively, and the remaining species of beetles used 3–4 host species. Table 1 shows the frequency of emerged beetles and their sex for each species of host plant. The body weights of the beetle species had interquartile ranges from 0.45 to 2.25 mg between species and significant differences were observed. The less heavy species was *M. cephalotes* and the heaviest was *M. porosus* followed by *M. cubicus*. The heaviest beetles emerged from the seeds of *M. aegyptia*, followed by the beetles emerged from seeds of *I. nil* and *I. hederifolia*, and the lightest beetles emerged from *I. triloba* (Table 2).

**Table 1:** Frequency of individuals of the species of *Megacerus* emerged by sex (female/male) and by host plant species.

Beetle species	Host plant species			
	*Ipomoea triloba*	*Ipomoea hederifolia*	*Ipomoea nil*	*Merremia aegyptia*
*M. cephalotes*	20/26			
*M. incisithorax*	4/17	5/26	0/8	
*M. flabelliger*		16/11		
*M. cubiculus*	21/1	30/2	3/1	0/1
*M. tricolor*		1/0	5/12	0/1
*M. cubicus*		3/5	93/80	18/9
*M. porosus*				209/185
Seed weight	7.1 ± 3.2 d	16.1 ± 4.3 c	25.2 ± 13.4 b	39.9 ± 8.9 a
Seeds collected	2362	1997	3510	2641

Mean (±SD) of seed weight (mg) by host plant species is showed, as well the total number of seeds (n) collected. Different letters denote significant differences between plant species (Tukey test, *P* < 0.05).

**Table 2:** Mean (±SD) of body weight (mg) by sex (female/male) and by host plant of the *Megacerus* species studied. In the cases with n = 1 the unique value is presented.

Megacerus	*Ipomoea triloba*	*Ipomoea hederifolia*	*Ipomoea nil*	*Merremia aegyptia*
*M. cephalotes* ^a^	0.61 ± 0.16/0.56 ± 0.17			
*M. incisithorax* ^*b*^	0.55 ± 0.17/0.61 ± 0.17	0.96 ± 0.11/0.82 ± 0.24	NA/1.1 ± 0.31	
*M. flabelliger* ^cb^		0.93 ± 0.25/0.81 ± 0.19		
*M. cubiculus* ^c^	0.65 ± 0.16/0.7	1.15 ± 0.3/0.9 ± 0.56	1.07 ± 0.29/0.8	NA/1.2
*M. tricolor* ^d^		0.6/NA	1.68 ± 0.36/1.37 ± 0.53	NA/1.5
*M. cubicus* ^d^		1.23 ± 0.55/1.34 ± 0.51	1.64 ± 0.39/1.36 ± 0.32	2.02 ± 0.26/1.66 ± 0.17
*M. porosus* ^e^				2.16 ± 0.42/2.08 ± 0.43

NA = no data. Different superscript letters denote significant differences in the average of the body weight at the level of beetle species (Tukey test, P < 0.05).

**Figure 3:**
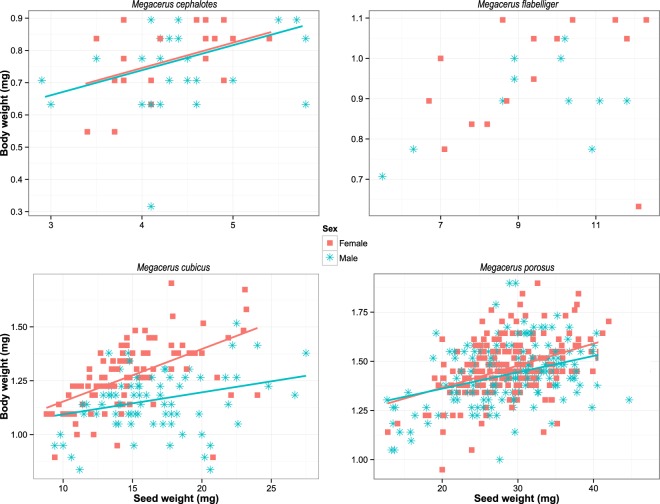
The relationship between seed weight and body weight in regard to the beetle sex in four species of *Megacerus*. Sex-biased regression lines are showed for each species (P < 0.05). In *M. cephalotes* and *M. falbelliger* there is not differences between the sexes, but in contrast, in *M. cubicus* and *M. porosus* sexes are different in the body weight scaling, being the females heavier than the males (see Table 3).

The comparison analysis of body weight across sexes within species of *Megacerus* was conducted for the species of beetles with enough data: *M. cephalotes*, *M. flabelliger*, *M. cubicus* and *M. porosus*. In general, the body weight both of females and males tends to increase with the seed weight, except for in the individuals of *M. flabelliger* with marginally not significant effect of seed weight on body size (Table 3). Differences in body weight between females and males were significant only in *M. porosus* (Table 3; Fig. 3). In fact the females of *M. porosus* are the heaviest individuals, both within and between species (Table 2). Significant interaction between sex and seed weight was observed only for *M. cubicus* (Table 3), being the females the sex with higher increments of body size in regard to the seed weight (Fig. 3). *Megacerus cephalotes* and *M. flabelliger* did not show significant differences between males and females, even though the average tendency across species is towards heavier females (Table 2).

**Table 3:** Effects estimated with the linear mixed model of the importance of the seed weight in regard to the beetle sex on the weight of body of beetles for combinations of species of *Megacerus* and species of host seeds with >10 data in each sex within host plant species.

Combination beetle × host seed	Source	Type II/III Sum of Squares	df	F	*P*
*Megacerus cephalotes* in *Ipomoea triloba*	Seed weight	0.075	1	5.851	0.0199
	Sex	0.017	1	1.359	0.2503
	Seed weight × Sex	0.014	1	1.121	0.2958
	Residuals	0.539	42		
*Megacerus flabelliger* in *Ipomoea hederifolia*	Seed weight	0.047	1	3.141	0.0896
	Sex	0.022	1	1.456	0.2397
	Seed weight × Sex	0.001	1	0.067	0.7981
	Residuals	0.347	23		
*Megacerus cubicus* in *Ipomoea nil*	Seed weight	0.659	1	39.127	<0.0001
	Sex	0.014	1	0.855	0.3564
	Seed weight × Sex	0.110	1	6.550	0.0114
	Residuals	2.850	169		
*Megacerus porosus* in *Merremia aegyptia*	Seed weight	1.263	1	69.969	<0.0001
	Sex	0.091	1	5.041	0.0253
	Seed weight × Sex	0.027	1	1.536	0.2159
	Residuals	7.041	390

**Figure 4:**
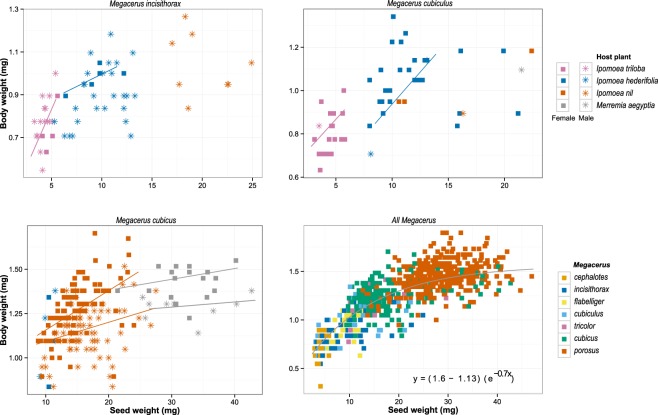
The relationship between seed weight and body weight in three species of *Megacerus* in regard to different host’s species. Significant regression lines are showed for each host (P < 0.05). In the three species, differences between hosts were observed. For the same *Megacerus* species, the heaviest hosts produced the heavier beetles (see Table 4). In the panel “All *Megacerus*” the overall relationship (the asymptotic equation is shown) between host seeds and beetles has shown pooling all the species.

The analysis of variance with mixed models to test the effect of different host seed species on the body weight of individuals within species of *Megacerus* was conducted independently for *M. incisithorax*, *M. cubiculus*, and *M. cubicus*. Results showed differences in the body weight with respect to the host species (Table 4). Coincidentally, the differences observed in the body weight of the beetles were proportional to the differences in seed weight between plant species (Table 1). Individuals of *M. incisithorax* that emerged from *I. triloba*, with smaller seeds, had lower body weight than the individuals emerged from *I. hederifolia*, with larger seeds. Individuals of *M. cubiculus* that emerged from *I. triloba* were less heavy than individuals emerged from *I. hederifolia*. Individuals of *M. cubicus*, one of the heaviest species, emerged from seeds of *I. nil* were less heavy than individuals emerged from *M. aegyptia*, the heaviest seed species (Table 2; Fig. 4).

**Table 4:** Effects estimated with the linear mixed model of the importance of the host plants and the beetle sex on the relationship between the seed weight and the weight of body of beetles. Analyses were conducted for *Megacerus* species with at least 40 data.

Seed beetle	Source	Type III Sum of Squares	df	F	*P*
*Megacerus incisithorax*	Host seed species	0.268	1	18.650	<0.0001
	Residuals	0.597	50		
*Megacerus cubiculus*	Host seed species	0.869	1	51.277	<0.0001
	Residuals	0.872	52		
*Megacerus cubicus*	Host seed species	0.390	1	20.181	<0.0001
	Sex	0.323	1	16.735	<0.0001
	Host seed species × Sex	0.002	1	0.116	0.7333
	Residuals	3.788	196

The comparison between the three regression models fitted to the data indicated that although the three models were statistically significant and had low values of residual standard error and a relatively adequate goodness of fit, the linear model had the poorest fit with respect to the quadratic and the asymptotic models (lineal: RSE = 0.16, r^2^ = 0.66, df = 811; asymptotic: RSE = 0.14, r^2^ = 0.74, df = 810; quadratic: RSE = 0.14, r^2^ = 0.74, df = 810). The last two models were similar in describing the rate of change in the body weight of beetles, although the asymptotic model was considered better because it more realistically described the weight ratio between the seeds and the beetles. The asymptotic model found that the general relationship between the seed weight and the beetle weight is proportional and linear at the lower values but reaches a top of change at the higher ones. Thus, an increase in the seed weight produces a proportional increase in the resulting beetle weight. However, the model show this proportionality decreases and fixes at some point of body weight value (1.6 ± 0.02 mg), and then subsequent increases in the seed weight will result in a slightly increase in the beetle. The relationship between the weights as well as the equation of the asymptotic model that describes it is represented in Fig. 4.

## Discussion

The body weight of an insect determines its life history, and in seed beetles, body weight is directly linked to the discrete amount of resources contained in the host seeds. Seeds are discrete units of food resources whose weight is a predictor of beetle weight, therefore predicts its performance in the adult life^2,6,28^. This phenomenon is widely documented for seed beetles and is also true for the group of *Megacerus* species studied here, i.e., in general, the seed weight of the Convolvulaceae host plants predicts the body weight of *Megacerus* adult beetles.

However, the results also show that differences in the scaling of body weight of beetles are associated with sexual dimorphism, by the type (species) of host seed, and probably by intrinsic limits in the body weight between beetle’s species.

Within the same species of beetle, the females have a greater scaling in body weight than the males. Besides, species of *Megacerus* that use different host species that differ in the weight of seeds have different scaling in the body weight. The increase in seed weight generally results in an increase in the corporal weight of the adult beetles, however, it halts at a threshold value of body weight, and after reaching this threshold, and heavier seeds will result in slightly increments of body weight.

In addition to the sexual dimorphism in the antennas and eyes, dimorphism in body weight was observed in two of the four species analyzed (*M. cubicus* and *M. porosus*) only, this probably because the relatively low number of data registered in the other two species (*M. cephalotes* and *M. flabelliger*). Despite of this, the general tendency in the average of body weight across *Megacerus* species in the assemblage studied here, is that females are slightly heavier than males. However, the sample size needs to be increased for the species having low numbers in order to extent the analysis to more species and produce statistically significant differences. Although this, results for the analyzed species are concordantly with other studies in relation to sexual dimorphism in insects. It was observed a bias towards females in body weight^8,9,29,30^ and attributable to the existence of a greater selection pressure towards female fecundity, with larger females presenting a higher number of ovarioles, laying more eggs to reach greater fertility^5,31,32^. In insects, females appear to be more sensitive to host quality than males so that in high-quality conditions there will be a larger relative increase in female than male size^33^.

The males of the species of *Megacerus* analyzed are smaller than females, however they present (as all *Megacerus* species) pectinate antennas and are highly elaborate in their morphology, thus denoting that males and females of the genus *Megacerus* have different investment of resources in reproduction. In females, fecundity is maximized, which leads to a consequent increase in body weight, whereas in males, external morphology characteristics are probably maximized to increase the probability of effective copulation^32^ but do not increase the size and body weight significantly.

The results also show that in *Megacerus* species that use more than one species of host seed, if the weight of the seed is lower, the beetles that emerge from it will have a lower body weight than those emerging from the host species with heavier seeds. This denotes that in the assemblage of species of *Megacerus* and Convolvulaceae, the type of host is an important factor in determining the body weight scaling. Because the relatively low sample size in most of the *Megacerus* species registered here, the effect of the type of host seed on the body weight was possible to observe only in species with adequate sample size: *M. incisithorax*, *M. cubiculus*, and *M. cubicus*. In the three species, the results were consistent in that the differences between hosts in the weight of seed are associated to the differences in the body weight between species of *Megacerus*, and for *M. cubicus*, this effect was even observed between sexes. The results are concordant with the observed in other bruchids species that use host species that differ in the seed quality^6,34^, and suggest the existence of plasticity in life-history traits in differing qualities of host seed species^35^.

From the plant perspective, the group of host seeds in this system represents a gradient of food availability for the larvae of different species of *Megacerus* as the host species differ in the seed weight. This gradient of food availability roughly corresponds to the differences between species of *Megacerus* in the body weight observed in the emerged beetles. At one end of the range are the lightest seeds with the least amount of food, and these seeds correspond to the *I. triloba* plant; thus, the lightest beetles, *M. cephalotes*, *M. incisithorax*, and the smallest individuals of *M. cubiculus* emerged from these plants. At the other extreme are the heaviest seeds from *M. aegyptia*; thus, the heaviest beetles, *M. porosus* and *M. cubicus*, emerged from these plants. These results give additional evidence to support the notion that the host quality determines the guest quality.

However, the proportional effect of the seed weight on the body weight of beetles can vary in its rate of change at the extreme of the range of values. By pooling all species of *Megacerus*, the analysis shows that the relationship between the seed weight and the body weight is consistently proportional until a certain threshold, after which heavier host seeds did not proportionally influence the body weight of the emerged beetle. The asymptotic regression predicts that in our data and the analysis of *M. cubicus* brings some evidence in this respect. Although, the mean of body weight was lower in individuals emerged from *I. nil* than in ones emerged from *M. aegyptia*, the range of variation decreased. Additionally, the heaviest seeds belonging to the *M. aegyptia*, whose seeds, weigh over 30 mg, produced beetles with body weights similar to that of beetles emerged from lighter seeds. For example, the maximum average of body weight was observed in the individuals of *M. porosus* 3.6 mg in a female of *M. porosus* that emerged from a seed weighing 32.6 mg (*M. aegyptia*), and the maximum seed weight of this plant was 47 mg, from which emerged a 2.1 mg beetle (male, *M. porosus*). One plausible explanation that needs to be corroborated is that the maximum observed body weight value for each beetle species can be considered a threshold weight, and after reaching it, there is no further gain in body weight, even when there is more food availability. If this is a correct explanation, the threshold weight is similar to the critical weight which is defined as the weight at which further growth is not necessary for metamorphosis^36^. Being reached the critical weight, the larva at its last instar initiates the metamorphosis. When the larva enters its last instar (fourth instar for *Megacerus*^18^), it acquires the greatest weight gain until reaching a critical weight value, after that, even when the larva has greater availability of food in its host seed, it will stop growing if the hormonal release of metamorphosis has begun^37^.

A characteristic aspect above mentioned of the assemblage of species *Megacerus*-Convolvulaceae studied here, is the apparently specialized distribution of beetle species across seed species. In general, not all species of *Megacerus* develop in all seed species of the four host plants studied, although they are differentially distributed in terms of the weight and size of the seeds. At one end of the range of weights and sizes are lighter and smaller species of *Megacerus* that only developing in seed species that are lightest and smallest. At the other end of the weight range are the heavier beetle species that only grow in the heavier seed species. However, the results cannot be explained by a niche differentiation^38^ via competitive exclusion mainly because the low frequency of infestation across host plants. A phenomenon involving female preferences can be a more plausive explanation. From setting any effects of competitors on performance aside, in taxa with relatively few hosts like *Megacerus*, the female’s preference in oviposition may be promoting specialization between the beetle species^39^. Because female beetles will evolve to oviposit on hosts on which their offspring fare best, a fine-tune differential distribution is expected to occur in *Megacerus* species on their hosts. In this work, three *Megacerus* species use a unique host species but the others use three different hosts.

In conclusion, the assemblage of species of *Megacerus* and their host seeds, a direct and proportional relationship is observed between the weight of the seed and the weight of the beetle; however it seems to reaches an asymptote value and subsequent increases in seed weight do not result in an increase in body weight. Differences in the body weight between the sexes are evident and female-biased, in an environment (host seed) that promotes the increasing of body weight mainly in females. Variations in the body weights of the seven studied species of *Megacerus* are associated with variations in the seed weight among host plants, which suggests a diet specialization not via competitive exclusion but due to beetle size and growth rate adaptations to an optimal or preferred host (female oviposition choice), with subsequent limitations on the ability to colonize suboptimal hosts. The results suggest that the diversification of *Megacerus* species, as granivores of the seeds of Convolvulaceae, is influenced by the weight ratio between seeds and beetles, and bounded by intrinsic characteristics of species of beetles and of seeds, that is promoting the species specialization in the assemblage of beetle-seed interactions studied here.
